# Motivated Interpretations of Deceptive Information

**DOI:** 10.3390/brainsci11030297

**Published:** 2021-02-26

**Authors:** Sigal Vainapel, Yaniv Shani, Shaul Shalvi

**Affiliations:** 1 Coller School of Management, Tel Aviv University, Ramat Aviv 6997801, Israel; sigal.vain@gmail.com; 2 Amsterdam School of Economics, University of Amsterdam, 1012 WX Amsterdam, The Netherlands; S.Shalvi@uva.nl

**Keywords:** moral judgment, lies, information seeking, behavioral ethics, dishonesty

## Abstract

We examine whether people seek information that might help them make sense of others’ dishonest behavior. Participants were told that a hypothetical partner (either a friend or a stranger) had engaged in a task in which the partner could lie to boost their earnings at the expense of the participant’s earnings. Participants were less likely to search for information that can justify potential dishonest behavior conducted by a friend than by a stranger (Experiment 1). When participants knew for certain that their partners had lied to them, they were less likely to assume that that the lie was justified when told that the partner was a friend rather than a stranger (Experiment 2). The results imply that people are more likely to search for information that may reduce the severity of possible dishonest behavior when a stranger, rather than a friend, is responsible for the behavior.

## 1. Motivated Interpretations of Deceptive Information

We want to believe that our friends are honest, especially when they interact with us. However, some situations may tempt even our closest friends to dishonestly promote themselves at our expense. Coworkers may take more credit than they deserve for a joint project; a partner avoids admitting that a new relationship is developing or conceals a separate bank account; a friend says she will pass on a resume at her workplace and put in a good word but does not. The current study investigates whether people want to know whether friends (or strangers) lied to them. More specifically, we ask whether people search for justifications that explain others’ dishonest behavior—information that can potentially mitigate the severity of the lie. Two experiments suggest that people do search for and generate information that might enable them to make sense of why others have lied to them. Notably, however, this tendency is moderated by the relationship between the deceiver and the deceived and their motivation to interpret the information retrieved as a justified lie. 

## 2. Justifications Shape People’s Lies 

There are obvious economic benefits to lying. Yet, people are generally aversive to telling lies and try to avoid behaving dishonestly, even when the likelihood of getting caught is low [1,2,3]. People allow themselves to lie as long as they can feel that they are honest individuals and that they can be perceived as such [4,5], i.e., when they can explain to themselves that their minor lies do not project dishonesty [3,5,6]. Thus, an important mechanism that facilitates dishonesty is one’s ability to justify a lie. Self-serving justifications enable people to do wrong but feel moral; thus, the availability of justification determines the magnitude of a person’s lies [7,8,9,10,11]. 

As a case in point, take the study reported in [9]. Participants rolled a die and received money according to the outcome they reported rolling. Higher reported outcomes meant higher payoffs. When participants rolled the die three times and reported the outcome of the first roll only, they lied more compared to participants who rolled only once. Analyses of the patterns of reported outcomes suggested that participants reported the highest of the three values they saw, instead of the outcome of the first roll (see also [8,10,12,13]). Observing a more desirable outcome on the second or third roll compared to the first roll that counted for pay liberated people to use this outcome to craft their lies. Not only do people find it easier to lie when they can justify doing so, they also consider justified lies to be less dishonest compared with unjustified lies [9,14,15]. 

## 3. Dishonesty and the Brain 

Note that not only that justified lies mitigate the perceived severity of the lies; neuro functioning may cause them to also experience and code justified lies differently than unjustified lies, depending on their values [16,17], social roles, and motivation [18]. For instance, participants playing for the opportunity to achieve real monetary gain while undergoing an fMRI procedure (by reporting their predictions for the outcome of a randomized coin flip and compensated for accuracy) showed a strong prefrontal activity in response to opportunities to achieve gain dishonestly. In response to anticipated dishonest responses, individuals showed an increase in their nucleus accumbens activity, which connects between the limbic system and reasoning processes. Importantly, there was also an increase in the dorsolateral prefrontal activity that is known to decrease the effect of dishonesty concerns in economic games when they collide with self-interest motives [16]. Similarly, recent research, allowing spontaneous cheating during fMRI scanning, found as well that the neural mechanism that enables people to cheat yet perceive themselves as honest is located in nucleus accumbens. According to the findings, activity in the nucleus accumbens encourages cheating for people who cheat a lot. Interestingly, neural activities that are associated with self-control (anterior cingulate cortex and inferior frontal gyrus) encouraged honest behavior for dishonest participants yet encouraged cheating in honest participants [17]. These findings suggest that neural mechanisms are indeed associated with emotional responses and behavior of individuals cheating and behaving unethically, yet the responses are also dependent on the moral values of the cheating individuals and motivation to behave unethically.

## 4. Information Search 

What kind of information do people search when they suspect dishonesty? Since justifications can mitigate the perceived severity of lies, people might be interested in searching for information about available justifications when they know or suspect they have been lied to. Overall, people dislike the feeling of uncertainty and continuously seek to resolve uncertainties in their lives [19,20,21]. A common way to reduce uncertainty is to obtain information. In fact, the drive to seek information is so strong that people sometimes search for information that is painful and irrelevant for future use [22,23,24,25,26]. For instance, people search for information about the winning numbers of a lottery ticket that they did not send out, even though there is nothing they will be able to do with the information, which may only cause them discomfort [24]. It is important to note that people search for such information not because they welcome exposing themselves to unpleasant experiences but because the state of ignorance is in itself disconcerting [24,26,27,28].

Definite knowledge, even when painful, may both bring closure and teach the information seeker to be more careful next time. However, there are certain factors that encourage individuals to maintain ignorance rather than search for information. One such factor is the avoidance of negative arousal and rumination [24]. Shani, Van de Ven, and Zeelenberg [29] suggested that when facing a decision between learning new information and remaining ignorant (e.g., deciding whether to find out the results of an exam before going on a vacation), people choose the option they believe will best regulate their negative feelings. Taken together, these findings suggest that when information has the potential to cause us pain—for instance, information indicating that our friends are actually benefiting at our expense—people will search for such information if they believe it will make them feel better than staying ignorant [24,26]. 

Given that the existence of a justification for a lie can mitigate the extent to which people perceive the lie as dishonest, we suggest that people who have been lied to might seek to regulate their negative feelings about the lie by searching for information that can justify the lie and explain the misconduct of the liar. Yet people will seek to expose themselves to such information only when they feel they can tolerate it. We suggest that tolerance for such information—and, accordingly, the tendency to search for information that might constitute justification for a lie—is affected by the relationship between the liar and the deceived (e.g., whether the two are close friends or strangers). 

## 5. Different Expectations from Friends and Strangers 

Expectations people hold are known to influence the judgment of one’s own and others’ behavior (e.g. [30,31,32]). People hold different expectations from their close friends than they do from strangers. Specifically, because our friends are valuable to us and reflect on us directly, we expect them to be more pro-social, fair, and honest than we expect strangers to be [33,34,35,36]. Not only do people hold high expectations from their friends, they also actively try to live up to these expectations in order to maintain their close relationships [37]. Correspondingly, when friends engage in a behavior that violates the expectations of them, they are judged negatively compared to strangers performing the same behavior [34,38,39,40,41,42]. For example, participants playing a competitive card game in which the deck of cards was fixed remembered that their friends made more competitive moves than strangers did, although the number of possible competitive moves was identical [43]. In addition, self-serving lies told by friends are judged as less acceptable than the same lies when told by strangers [44,45]. We suggest that the existence of different expectations for honesty from friends and strangers leads to different emotional costs of finding out that friends, as opposed to strangers, have lied. Therefore, the relationship between the liar and the deceived party can influence the extent to which the latter will seek information that may explain the lies. 

The question remains: Would people want to make sense of friends’ possible lies more than strangers’ lies by searching for further information regarding their wrongdoing? On the one hand, because a lie told by a friend can be extremely painful and harmful for the relationship, people may want to believe that if their friends are lying to them, at least there is a reason for them to do so—that there is a justification for the lie, rendering it less severe. It follows that as relationships with strangers are temporary and less emotional, people are expected to care less when they are lied to by strangers, making the search for justifications less necessary. We call this the justifying friends’ wrongdoing hypothesis. On the other hand, because people have high expectations from their friends, and because they perceive violations of these expectations as severe [33,34,39,41], people might not be willing to easily forgive a friend who lied to them. Since justifications can lessen the perceived dishonesty of a lie, making it easier to forgive it, people may not be inclined to seek information, suggesting that their friends’ lies could be justified. We call this the avoiding justifying friends’ wrongdoing hypothesis.

To test these hypotheses, we conducted two experiments. In each experiment, participants were asked to evaluate how they would behave in various scenarios in which a friend (vs. a stranger) lied to them or was suspected of lying. Specifically, they were provided with scenarios in which they were told that a hypothetical partner, either a friend or a stranger, had rolled a die and reported the outcome, impacting their own and participants’ outcomes (the more one profits, the less the other profits). In the first experiment, we examined how people evaluated the honesty of their partners’ reports regarding the outcomes of their die rolls. We further measured the extent to which people sought information about the outcomes their partners had actually obtained. The second experiment examined the extent to which a person who has been lied to is likely to assume that the liar had some justification for his or her behavior, given that the liar is either a friend or a stranger. 

### 5.1. Experiment 1

The first experiment examined whether people search for information to a different extent in order to clarify a potential lie by a friend vs. a stranger. 

Participants and procedure: Seventy-nine management students (47 males, *M*_age_ = 22.6) participated in the lab experiment for course credit. We employed a 2 (relationship: friend vs. stranger) × 6 (reported outcome: 1 vs. 2 vs. 3 vs. 4 vs. 5 vs. 6) design, with relationship as a between-subjects factor, and reported outcome as a within-subjects factor. 

Participants were given a hypothetical scenario where they engaged in a die-rolling task with a partner. Each participant was randomly assigned to one of the relationship conditions and was asked to imagine a partner described either as “a close friend of yours” (“friend” condition) or as “a stranger” (“stranger” condition). As a manipulation check, participants answered three questions assessing how close they felt to the imagined partner (i.e., “How close do you feel to the other participant?”; “How many times a week do you contact the other participant?”; “To what extent are you willing to share an embarrassing story of yours with the other participant?”; α = 0.93). 

The participants read the instructions of the task given to their imagined partner. The die-rolling task was described to the participant as follows: the participant’s partner has been instructed to privately roll a die three times and report the outcome of the first roll to determine both own and participant’s profit [9]. The first roll outcome their partner reported will influence both the partner’s and the participant’s profits. Specifically, the partner will receive a sum of money equal to the outcome of the first die roll, and the participant will receive $6 minus the die-roll outcome the partner reported. For example, if the partner reports a die-roll outcome of 4, the partner will receive $4, and the participant will receive $6 − $4 = $2). Four questions examined the comprehension of the task. If a participant failed to answer all the questions correctly, an experimenter explained the task to him or her again, and the participant had to retake the comprehension questions (see full scenario and comprehension checks in Supplementary Materials). All participants in this study correctly answered the comprehension questions and no participants were removed from the study. Then, participants were presented with six die-roll outcomes (1 to 6) supposedly reported by their partner in ascending order. Participants were not informed of the *actual* outcome of the first die roll (only the reported outcome) nor were they told the outcome of the second or third die roll. For each reported-outcome, the participants were asked to do the following: (1) estimate what were the partner’s actual first and second die-roll outcomes; (2) rate the extent to which they believed that the outcome reported by their partner was a lie; (3) indicate the extent to which they wanted to know what their partner’s second die-roll outcome was; and (4) indicate how curious they were and the extent to which they wanted to seek information about their partner’s *second* die-roll outcome. Ratings for the latter three items were on a 7-point scale (1 = not at all; 7 = very much).

#### 5.1.1. Results 

Manipulation check: Participants reported feeling closer to the partner when that person was described as a close friend (*M* = 5.73, *SD* = 1.06) than as a stranger (*M* = 3.46, *SD* = 2.12), *t*(77) = 6.00, *p* < 0.001. 

Estimated first roll outcome: In order to assess perceptions of (dis)honesty, we coded whether participants estimated that their partner’s actual outcome was equal to the reported outcome (reflecting an honest report) or whether it was unequal to the reported outcome (reflecting a dishonest report). Note that although participants reported a specific evaluation of their partner’s reported outcome, we only report whether their evaluations were equal or unequal to the reported outcome in order to avoid over-interpreting the strength of the dishonest report. Chi-square tests were used to assess the effect of relationship condition on participants’ perceptions of their partners’ dishonesty. The analysis revealed that when the reported outcome was low (1, 2, and 3), participants in the friend condition did not differ from those in the stranger condition in their assessment of the likelihood that the partner was lying, all *p’s >* 0.41. Consistently, when participants were presented with these low outcomes, 90% of their responses indicated that they believed the partner had reported the outcome honestly. However, when the reported outcome was high (4, 5, and 6), participants who were told their partner was a stranger were more likely to estimate the report to be a lie compared with participants who were told their partner was a friend. Specifically, when the reported outcome was 4, 22.5% of participants in the stranger condition estimated that the report was a lie, whereas no participants in the friend condition estimated that the partner was lying, *χ*^2^(1,*n* = 79) = 9.03, *p* = 0.002. Similar patterns emerged when the reported outcome was 5 (stranger condition: 30% vs. friend condition: 5.1%; *χ*^2^(1,*n* = 79) = 8.38, *p* = 0.004), and 6 (stranger condition: 35% vs. friend condition: 7.7%, *χ*^2^(1,*n* = 79) = 8.72, *p* = 0.003). 

**Figure 1:**
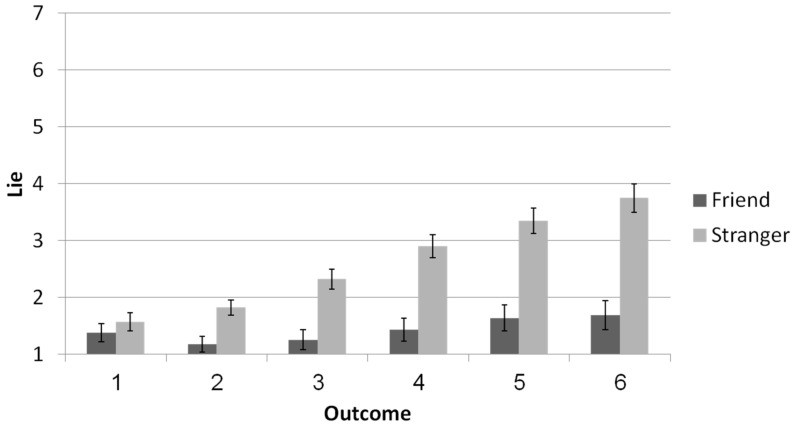
Perceived lie as a function of interaction partner and outcome (Error bars are +1 SE around the mean).

Is it a lie? We employed an ANOVA with relationship (friend vs. stranger) as a between-subjects variable and reported outcome (1, 2, 3, 4, 5, and 6) as a within-subjects factor, predicting the extent to which participants perceived their partners’ reports to be a lie; see Figure 1. The analysis revealed a main effect for relationship: Overall, participants judged strangers’ behavior as more dishonest (*M* = 2.62, *SD* = 1.46) compared with friends’ behavior (*M* = 1.43, *SD* = 1.44), *F*(1,77) = 32.59, *p* < 0.001, *η_p_*^2^ = 0.30. In addition, higher reported outcomes lead to higher dishonesty ratings *F*(1,77) = 50.77, *p* < 0.001, *η_p_*^2^ = 0.40. The effect was qualified by an interaction between relationship type and reported outcome, *F*(177) = 23.17, *p* < 0.001, *η_p_*^2^ = 0.23. Simple effects analyses of the differences between strangers’ and friends’ suspicions for every reported outcome independently revealed that participants were more suspicious of strangers than of friends for all reported outcomes except 1 (*p* = 0.39 for a reported outcome of 1; *p* < 0.01 for all other reported outcomes).

**Figure 2:**
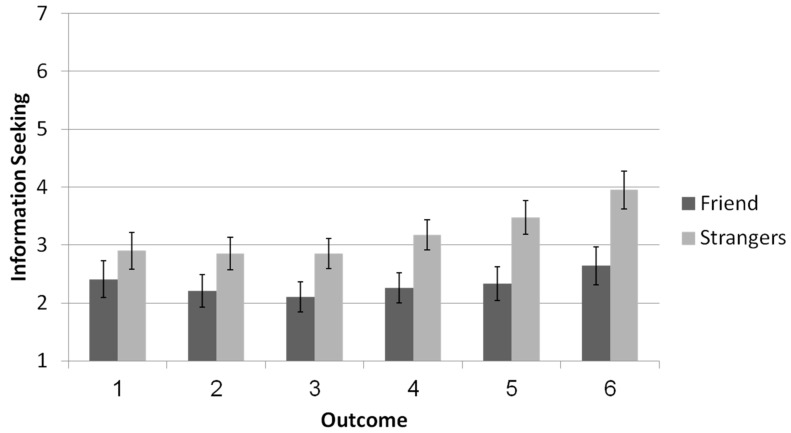
Information search about the second toss as a function of interaction partner and outcome (Information search was calculated by averaging participants’ reports on a curiosity and wanting to know questions. Error bars are +1 SE around the mean).

Information seeking (see Figure 2)*:* Information seeking was calculated by averaging participants’ responses to the questions about curiosity and wanting to know the outcome of the second roll. An ANOVA with relationship (friend vs. stranger) as a between-subjects variable and reported outcome (1, 2, 3, 4, 5, and 6) as a within-subjects factor, predicting the extent to which participants wanted to seek information about the outcome of the second roll outcome revealed a main effect for relationship. Participants were more likely to search for information about their partners’ second-roll outcome when their partner was a stranger (*M* = 3.22, *SD* = 1.81) compared with a friend (*M* = 2.36, *SD* = 1.75), *F*(1,77) = 5.72, *p* = 0.019, *η_p_^2^* = 0.07. A main effect was also found for the reported outcome (1 to 6) *F*(1,77) = 8.41, *p* = 0.005, *η_p_^2^* = 0.10. Simple effects analysis indicated that participants were less likely to search for information on the second roll when the reported outcome was low (i.e., an outcome of 1, 2, or 3) than when the reported outcome was high (i.e., an outcome of 4, 5, or 6); *p <* 0.001. A marginally significant interaction effect was observed for reported outcome and relationship, *F*(1,77) = 3.05, *p* = 0.085, *η_p_*^2^ = 0.04. 

#### 5.1.2. Discussion

The results suggest that the higher the reported outcome was, the more people suspected that the person reporting the outcome had lied. People were also more suspicious toward strangers than they were toward friends. Consistently, participants were more likely to search for information that might justify a possible lie when interacting with strangers than with friends (i.e., what was the outcome of the second roll). 

These findings support the avoiding justifying friends’ wrongdoing hypothesis, whereby people are less likely to search for information justifying a lie told by a friend than by a stranger, possibly because they are unwilling to accept that a friend is lying to them. However, some alternative explanations may exist for our findings. First, people might not suspect their friends of lying to them (as Experiment 1’s findings also suggest). Accordingly, if they believe their friends are honest, they have no reason to search for information that can justify a lie that did not even occur. Second, it has been shown that the presence of justification enhances the possibility that a person will lie [8,9]. Thus, people may actively avoid information that can justify a friend’s lie, because the existence of such information might intensify the suspicion that the friend did, in fact, lie. A third option relates to the fact that searching for justifying information does not always yield the desired results. Specifically, it is possible that people actually do want to learn that friends have justifications for lying but do not want to search for such information because of the possibility that they might be disappointed if they find out that, in fact, the lie is not justified. 

In Experiment 2, we asked participants to estimate possible outcomes of a third die roll of either a friend or a stranger who lied to them. As in this case, the lie was confirmed and not merely suspected, we were able to test whether the avoidance of justifying information when suspecting friends is due to unwillingness to justify friends’ dishonesty and not due to confounding factors related to suspicion of the friend (the first and second alternative explanations). In addition, Experiment 2 enabled us to test the third alternative explanation. If avoidance of information about a friend’s lie is motivated by the fear of finding out that the lie was unjustified, the person who has been lied to is likely to estimate that the friend had some way to justify the lie. Conversely, if people avoid information because they are unwilling to accept a friend’s dishonesty, they are unlikely to estimate that their friend had a way to justify the lie. 

### 5.2. Experiment 2

Participants were given the same scenario as in Experiment 1 with one main difference: they were informed about the actual outcomes of their partners’ first and second die rolls in the task. In addition, instead of asking participants whether they would have liked to learn the second outcome of their partners (i.e., search information), in Experiment 2, we asked them to estimate the outcomes of their partners’ third die roll. This enabled us to assess whether they believed that their partners had justifications for their lies. 

*Participants and procedure:* Sixty-seven management students (34 males, *M*_age_ = 22.1) participated in the lab experiment for course credit. We employed a 2 (relationship: friend vs. stranger) × 3 (reported-outcome type: justified dishonest report vs. unjustified dishonest report vs. honest report) × 2 (gap size between reported outcome and actual outcome: small gap vs. large gap) design, with relationship as a between-subjects variable, and we reported-outcome type and gap size as within-subject factors. 

As in Experiment 1, participants were asked to imagine engaging in a die-rolling task with a close friend or a stranger. As a manipulation check, we asked participants to rate how close they felt to the other person (three questions as in Experiment 1, α = 0.92). Participants learned the other person’s reported first die-roll outcome (as in Study 1) as well as the actual outcomes of the first and second die rolls. This allowed participants to assess whether the person they interacted with lied or was honest. 

Participants had to evaluate 18 reported outcomes that were provided by the other person: six reported outcomes were honest (i.e., the other person reported correctly the outcome that appeared on the first die roll); six were justified lies (i.e., the other person reported a higher value than the outcome seen on the first roll but matched the value appearing in the second roll); and six were unjustified lies (i.e., the other person reported a higher value than the outcomes of both the first and second rolls). Note that an unjustified lie could potentially be justified by the third outcome of the third die roll, which was not provided to participants. Among justified and unjustified lies, we varied the magnitude of the lies so that in some cases there was a large gap (of 3 numbers) between the actual and reported outcomes (e.g., reported first roll outcome = 6, actual first roll outcome = 3), and in other cases, there was a small gap (of 1 number; e.g., reported first roll outcome = 6, actual first roll outcome = 5). After seeing each set of outcomes, participants were asked to evaluate the following on a 7-point scale (1 = not at all; 7 = very much): (1) the extent to which they considered each report to be a lie and (2) the likelihood they would have acted similarly if they were in their partner’s position. 

Most importantly, participants were asked to estimate the outcome of the third roll. The estimation enabled us to assess whether participants believed their partners had justifications for their lies. If a participant estimates that the third outcome was the same as the partner’s reported outcome, when the reported outcome is a lie, we can infer that the participant assumed the other person had a justification for lying. As in study 1, all participants in this study correctly answered the comprehension questions and no participants were removed from the study.

#### 5.2.1. Results 

Manipulation check: A *t*-test with relationship condition (friend vs. stranger) as the independent variable and sense of closeness as the dependent variable confirmed that the manipulation was successful: Participants perceived the hypothetical partner as closer when that person was described as a friend (*M* = 5.98, *SD* = 0.95) than when the person was described as a stranger (*M* = 3.47, *SD* = 2.09), *t*(65) = 6.37, *p* < 0.001.

**Figure 3:**
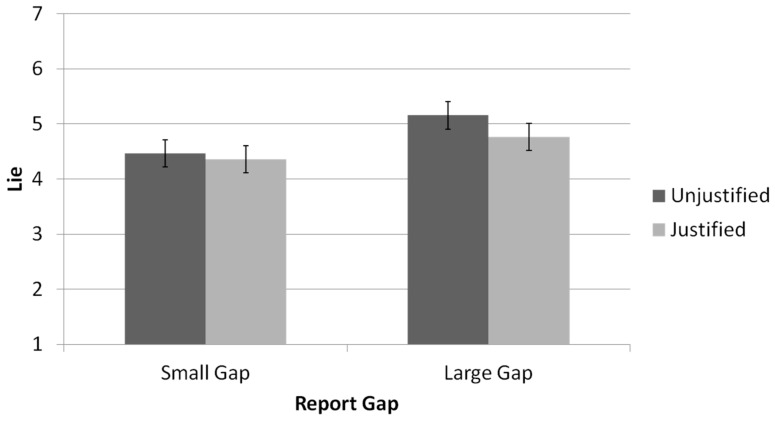
Perceived lie as a function of the gap between real and reported outcome and available justification (Results are across the both interaction partner conditions. Error bars are +1 SE around the mean).

Is this a lie? As in past work [14], we focused on comparing justified to unjustified lies. We employed a 2 (relationship type: friend vs. stranger) × 2 (reported-outcome type: justified lies vs. unjustified lies) × 2 (gap size: small gap vs. large gap) repeated-measures ANOVA (see Figure 3). Justified lies (i.e., untruthful reports equal to the second-roll outcome) were considered as less dishonest (*M* = 4.56, *SD* = 2.02) compared with unjustified lie (untruthful reports unequal to the second roll outcome; *M* = 4.81, *SD* = 2.02), *F*(1,65) = 8.39, *p* = 0.005, *η_p_*^2^ = 0.11. A main effect was found for gap size, showing that a large gap between the actual and the reported outcomes led to a higher perception of dishonesty (*M* = 4.96, *SD* = 2.03) compared with a small gap (*M* = 4.41, *SD* = 2.06), *F*(1,65) = 32.54, *p* < 0.001, *η_p_*^2^ = 0.33. A marginal interaction effect between reported outcome and gap size, *F*(1,65) = 3.08, *p* = 0.084, *η_p_*^2^ = 0.05, was found as well. No main effect was found for the relationship type, *F*(1,65) = 0.35, *p* = 0.557, and no interactions were found between relationship type and dishonest report or gap size (*p* > 0.1 in all cases); lies told by friends were perceived as similarly dishonest to lies told by strangers. 

**Figure 4:**
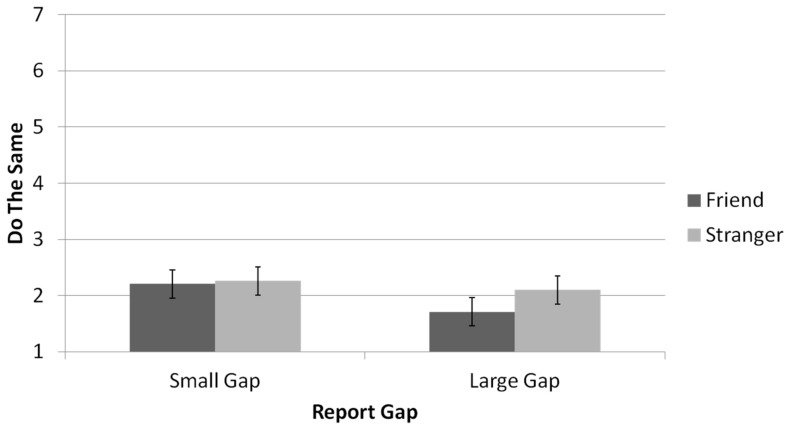
Participants’ statements they would have done the same as the interaction partner, as a function of the gap between real and the interaction partner (Results are across the both justification conditions. Error bars are +1 SE around the mean).

Would you do the same? A main effect was found for reported-outcome type, showing that participants were more inclined to report they would have done the same (i.e., lie) when the lie was justified (*M* = 2.15, *SD* = 1.44) than when the lie was unjustified (*M* = 1.99, *SD* = 1.29), *F*(1,65) = 4.74, *p* = 0.033, *η_p_*^2^ = 0.07; see Figure 4. Additionally, participants were more willing to lie if the gap between the reported outcome and the real outcome was small (*M* = 2.23, *SD* = 1.45) than if the gap was large (*M* = 1.91, *SD* = 1.27), *F*(1,65) = 13.37, *p* = 0.001, *η*^2^ = 0.17. The interaction between relationship type and gap size, *F*(1,65) = 3.45, *p* = 0.068, *η_p_*^2^ = 0.05, was analyzed using a simple effects analysis, which revealed that participants in the friend condition were more likely to report that they would have behaved similarly to their partners (i.e., lied) when the gap was small (*M* = 2.21, *SD* = 1.43) than when the gap was large (*M* = 1.17, *SD* = 0.96), *p* < 0.001. Among participants in the stranger condition, the likelihood to behave similarly to the partner and lie was not affected by the magnitude of the gap between the observed outcome and the reported outcome (*M*_small_ = 2.26, *SD*_small_ = 1.49; *M*_large_ = 2.10, *SD*_large_ = 1.52), *p* = 0.211. 

Estimation of the third outcome: Participants were asked to indicate what they believed the outcome of the third die roll was. In the scenario, partners were asked to roll the die three times, but we reported only the first two outcomes. Asking participants to estimate the third outcomes allowed us to assess the extent to which participants “justified” their partners’ lies (i.e., estimated that the outcome of the third die roll is equal to the outcome reported by the partner). We counted the number of times participants indicated that they thought the outcome of the partner’s third die-roll outcome was equal to the value this person reported rolling on the first roll. For every participant, we measured the mean number of justification she or he estimated the partner had. 

**Figure 5:**
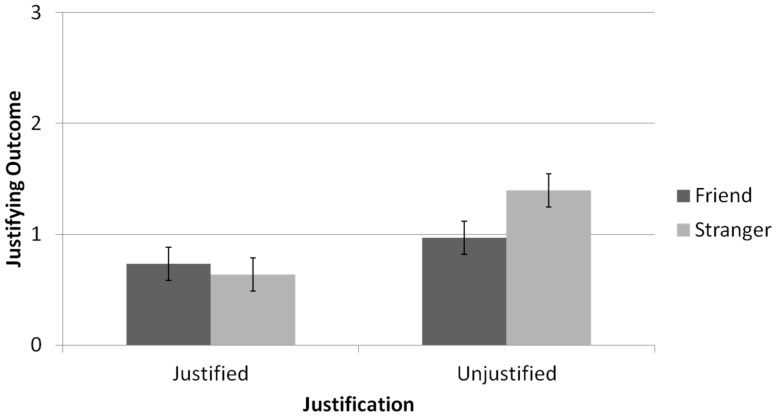
Justifications generated as a function of the interaction partner and already available justification (Results are across both report gap conditions. Justifications were measured by counting how many times each participants estimated the third die outcome to be equal to the reported first die outcome. Error bars are +1 SE around the mean).

The number of justifications (i.e., estimated third-roll outcome = reported first-roll outcome) was used as a dependent variable in a repeated-measures ANOVA, with relationship type as a between-subjects variable, and reported-outcome type and gap size as within-subjects variables; see Figure 5. The analysis revealed that participants were more likely to estimate that their partner had justifications when the reported outcome was an unjustified lie (*M* = 1.18, *SD* = 1.03) than when it was a justified lie (*M = 0*.69, *SD* = 1.73), *F*(1,65) = 18.93, *p* < 0.001, *η_p_*^2^ = 0.23. In addition, participants estimated their partner had more justifications when the gap between the actual and reported outcomes was small (*M* = 1.06, *SD* = 0.99) than when it was large (*M* = 0.81, *SD* = 0.91), *F*(1,65) = 10.06, *p* = 0.002, *η_p_*^2^ = 0.13. Participants in the friend condition did not significantly differ from participants in the stranger condition in the extent to which they estimated that their partners’ had justifications for lying (*M*_friend_ *=* 0.85, *SD* = 0.92; *M*_stranger_ *=* 1.02, *SD* = 0.96), *F*(1,65) < 1, *n.s.* However, a significant interaction effect was found between the relationship type and the reported-outcome type, *F*(1,65) = 5.24, *p* = 0.025, *η_p_*^2^ = 0.08. Simple effects analysis revealed that when the lie was unjustified, participants in the stranger condition were more likely to estimate that their partner had justifications for the lie compared with participants in the friend condition (*M*_stranger_ *=* 1.4, *SD* = 1.02; *M*_friend_ *=* 0.97, *SD* = 0.99), *F*(1,65) = 3.84, *p* = 0.05, *η_p_*^2^ = 0.06. In contrast, when the lie was already justified, participants in the two relationship-type conditions were equally likely to estimate that their partner’s lies were justified (*M*_stranger_ *=* 0.64, *SD* = 0.89; *M*_friend_ *=* 0.74, *SD* = 0.85), *F*(1,65) = 0.29, *n.s*.

#### 5.2.2. Discussion

The results show that participants estimated more justifications for their partners’ lies (i.e., were more likely to estimate that the outcome of the third die roll was equal to the partner’s reported outcome), when the lies were unjustified (by the second die roll) than when the lies were already justified. More interestingly, participants were more likely to estimate that their partners’ lies were justified when interacting with strangers than when interacting with friends. This means that participants were more likely to believe that strangers’ lies were justified (i.e., that they had actually received their reported outcomes on the third die roll) than to believe that friends’ lies were justified. 

Since participants in Experiment 2 knew with certainty that they had been lied to, the experiment enables us to rule out the alternative explanations put forward for the results of Experiment 1, in which participants could only suspect that they had been lied to: namely, that participants avoid information about their friends’ lies because (1) they do not, in fact, suspect their close friends of lying; (2) they wish to refrain from revealing that their friends have lied; or (3) they fear discovering that a friend’s lie is not justified. 

Notably, in Experiment 2, participants were more likely to assume that strangers’ lies were justified than to assume that friends’ lies were justified, even though the assumption of such justification might have helped participants to make sense of their friends’ dishonest behavior. As in Experiment 1, these findings support the avoiding justifying friends’ wrongdoing hypothesis. 

The results also suggest that not all lies are perceived similarly. When judging a lie, people take into consideration the size of the lie; in this experiment, participants did so by assessing the gap between the outcome of the first die roll and the reported outcome, which determined each partner’s payment. Small gaps between actual and reported outcomes (which are more easily justified) were considered to be less dishonest compared with large gaps, and participants indicated that they would be more willing to lie when the gap is small. These results support the notion that people find small lies to be less dishonest, and they support findings showing that people lie “just a bit” [5]. Moreover, lies that have some justification—in our case, reported outcomes that are equal to the actual outcome of the second die roll, which was not directly relevant for pay—are perceived as more honest than unjustified lies. In addition, when asked about their own willingness to report dishonestly, participants indicated that they would be more willing to lie when they could justify doing so. Lastly, the results show that lies told by friends and lies told by strangers are perceived as dishonest to a similar degree. 

## 6. General Discussion

In two experiments, we have shown that people’s responses to being deceived in a task by a friend are different from their responses to being deceived by a stranger. In Experiment 1, a scenario in which individuals have the opportunity to profit from dishonesty at the expense of others, we found that people might suspect strangers of doing so but possibly do not suspect friends. We also found that when imagining the possibility of having been lied to, people are more likely to search for information that might justify the lie (exposing the second die-roll outcome in the task) when the potential liar is a stranger rather than a friend. Results from Experiment 2 reveal that people consider justified lies to be less dishonest than unjustified lies. Moreover, despite the fact that people consider lies told by strangers to be just as dishonest as lies told by friends, they are more likely to assume that strangers’ lies are justified than to assume that friends’ lies are justified. 

These results suggest that the nature of a person’s relationship with a dishonest partner affects his or her motivation to interpret a situation as dishonest or not: people do not want to think their friends have justifications for lying. In turn, this motivation influences the extent to which the deceived party searches for (Experiment 1) or generates (Experiment 2) justifying information, which can reduce the perceived severity of the partner’s lies. It seems counterintuitive that people do not want to learn that their friends had justification when lying to them, considering the fact that close friends are usually the ones we interact with the most. Discovering a friend’s dishonest behavior is known to be particularly painful simply because such behavior violates norms of friendship and may put the relationship at risk [39,40,41,44,45]. Thus, one might expect that people would want to believe that their friends’ lies are justified, as such justification can reduce the severity of the lie [3]. However, our findings suggest that people are more likely to believe that strangers’ lies are justified than to believe that friends’ lies are justified. This observation supports the *avoiding justifying friends’ wrongdoing* hypothesis, which is based on the idea that because a lie told by a friend violates expectations of honesty, people are reluctant to forgive such behavior. The act of searching for information that can justify a lie constitutes an active effort to mitigate the dishonesty and severity of this behavior and is consistent with past research suggesting that the negative emotions arising from being mistreated by a friend can drive people to avoid engaging in such efforts [22,23,24,25,26]. 

Retaining close relationships is important for individuals. Yet, at some level, people avoid information that can justify unethical behavior by friends and is therefore likely to damage their close relationships. On the one hand, we would like to see dishonest behavior contained, and therefore, an uncompromising approach toward friends’ unethical behavior should be viewed as constructive. On the other hand, justifying friends’ minor wrongdoings can be useful for maintaining positive relationships. For example, a person may justify a coworker’s claim of extra credit for a joint project but not if this person is a friend, thus possibly harming the working relationship. Therefore, the high standards that people set for their friends might also have negative effects [43].

Our findings are consistent with past literature showing that people are willing to expose themselves to potentially painful information, even when the information is not relevant for future decisions [24,26]. Interestingly, our findings add to past research about the information search and avoidance strategies that individuals may utilize in order to regulate their feelings. Specifically, we suggest that people not only search for possibly painful information in order to alleviate uncertainty [24,28,29] but also may avoid potentially comforting information when interacting with friends who may have violated friendship norms. It is possible that the avoidance of justifying information reflects unwillingness to quickly forgive our friends for lying to us. Future research should further investigate and clarify the functional role of such information avoidance in the relationship between the deceiver and the deceived. In this regard, it should be particularly interesting to assess individuals’ personality traits that might influence the propensity to search for information about friends’ dishonesty, such as general trust [46] or the need for cognitive closure [26]. 

Although it is possible that people actively ignore information that can justify a friend’s misconduct, it is also possible that they are merely blind to such information. People demonstrate surprising blindness to information they do not want to know [47]. People can be blind to simple stimuli because they are preoccupied [48], to information that does not benefit them [49], to their own motivations to cheat [50], to their peers’ misconduct [51], and even to others’ intentions when presented with the outcomes of their behavior [52,53]. People see the reality they want to see; if a person believes a friend has betrayed them, they may be blind to information that can justify the friend’s behavior. More research is needed to determine whether the discounting of justifying information is intentional ignorance or unintentional blindness. 

Finally, consistent with past research [9,10], our findings suggest that people actually hold cognitive schemes about how people use information that can justify dishonest behavior in their favor. The fact that people search for information about the second and third die rolls of others they interacted with (i.e., justifications) suggests that people understand it is easier to provide false information regarding the outcome of a die roll after seeing higher outcomes in the rolls that followed. People seem to understand how others justify their lies by observing desired outcomes and even perceive justified lies as less severe than unjustified ones. 

An interesting direction for future research is to go beyond individuals’ decision processes and explore how people respond to information justifying a group’s misconduct. Past research has shown that people tend to cheat more when they work in a group context than when they work alone (e.g., [54,55,56,57]). However, it is less clear how people judge unethical behavior among individuals working together. Do people believe groups have justifications for their unethical behavior? Does a person’s tendency to search for information that might justify a group’s unethical behavior depend on whether the group is an in-group or an out-group? Finally, although past research vastly explored how justifications can mitigate the perceived severity of lies using hypothetical as well real incentives (e.g., [9,14,15]), future research may want to also replicate our findings about motivated interpretation of deceptive information between close friends and strangers, using incentivized decisions and not just hypothetical.

## 7. Summary

Although we want to believe that the people we interact with are honest, this is not always the case. It can be useful to learn how people deal with, and comprehend, situations in which others may have lied in order to benefit at their expense. We show that a person’s response to such situations—as reflected in their motivation to search for or avoid information that can justify a lie—can vary according to their relationship with the person who has potentially deceived them. Perhaps counter to intuition, people who have been lied to by friends are less motivated to believe that their partner had justifications for the lies, compared with people who have been lied to by strangers. Lying is an everyday behavior, occurring at the individual level between romantic partners, friends, coworkers, and more. By avoiding possible explanations for the dishonest behavior of their friends, people might harm their most valuable relationships. Thus, understanding the mechanisms behind information processing of others’ dishonest behavior can have implications for personal as well as business interactions. 

## Data Availability

The data presented in this study are openly available in OSF, doi:10.17605/OSF.IO/5RPTX.

## Supplementary Materials

The following are available online at https://www.mdpi.com/2076-3425/11/3/297/s1, Figure S1. Perceived lie as a function of interaction partner and outcome; Figure S2. Information search about the second toss as a function of interaction partner and outcome; Figure S3. Perceived lie as a function of the gap between real and reported outcome and available justification; Figure S4. Participants’ statements they would have done the same as the interaction partner, as a function of the gap between real and the interaction partner; Figure S5. Justifications generated as a function of the interaction partner and already available justification.
