# Supplementary material for: Motivated Interpretations of Deceptive Information
Source: Brain Sci. 2021 Feb 26;11(3):297. doi: 10.3390/brainsci11030297 (PMC7996790; doi:10.3390/brainsci11030297)
Supplement: Supplementary file 1 [file brainsci-11-00297-s001.pdf]

brain sciences

MDPI

Supplementary Materials

Motivated Interpretations of Deceptive Information

Sigal Vainapel, Yaniv Shani and Shaul Shalvi

Note: The study materials were originally in Hebrew.

1 Manipulation check for study 1 and study 2.

Please answer the following questions:

1. How close do you feel to the other participant? (From 1 "total strangers" to 7 "best friends")

2. How many times a week do you contact the friend [stranger] you were asked to imagine (phone calls, meetings, messages, etc.)? (From 1 "very few" to 7 "a lot").

3. To what extent are you willing to share an embarrassing story of yours with the friend [stranger] that you were asked to imagine? (from 1 "definitely would not share" to 7 "definitely would share")

2 Scenario for study 1 and study 2.

Now we will ask you to imagine that you are playing a game against the friend [stranger] that you imagined. First, please read the instructions that the other participant (friend [stranger]) read, and then answer four questions that verify you understood the task.

"For this part of the experiment, you will be given the opportunity to roll a single die to determine your pay. The procedure would be as follows:

Leaving the die under the cup, shake the cup back and forth vigorously to roll the die. Then, look through the hole in the cup to see what number you rolled. Do not remove the cup at any time. The number on the die (i.e., 1, 2, 3, 4, 5, or 6) is the number of dollars that you would earn for this part of the experiment.

Your outcome will also influence the pay of the second participant in the experiment. If you earn X dollars, the second participant will earn 6-X dollars. For example, if your outcome is 2, you will earn 2 dollars, and the other participant will earn 4 dollars.

After the first roll that determines your pay, we ask that you roll the die under the cup at least 2 more times so that you can verify for yourself that the die is legitimate. After rolling the die, you would write the number you got in the designated place. The outcome that you report will determine the number of dollars you and the other participant will get.

I rolled _____ in the first roll ".

These are the instructions the friend/stranger read.

3 Comprehension checks for study 1 and study 2.

1. Which roll determines the pay? (a. first roll, b. second roll)

2. If the other participant (the friend/stranger) rolled '1' in the first roll and '3' in the second roll, which number does the he/she needs to report according to the task rules? (a. 1, b. 3)

3. If the other participant (the friend/stranger) rolled a 6 in the first roll, how much money will you earn? (a. 0, b. 6)

4. If the other participant (the friend/stranger) rolled a 1 in the first roll, how much money will you earn? (a. 5, b. 1)

4 Study 1 dependent variables.

I think that the friend/stranger rolled:

First die roll: _________

Second die roll: _________

Please answer the following questions: (*from 1 'not at all' to 7 'very much'*)

1. To what extent do you believe that the reported (first) outcome is a lie?

2. To what extent would you want to know the outcome of the second die roll?

3. To what extent do you feel curious to about the outcome of the second die roll?

5 Study 2 presented outcomes.

1. Rolled 3 and then 2, reported 4. (*Unjustified lie)*
2. Rolled 3 and then 6, reported 6. (*Justified lie)*
3. Rolled 2 and then 1, reported 3. (*Unjustified lie)*
4. Rolled 2 and then 1, reported 2. (*Honest)*
5. Rolled 4 and then 3, reported 4. (*Honest)*
6. Rolled 2 and then 1, reported 5. (*Unjustified lie)*
7. Rolled 2 and then 5, reported 5. (*Justified lie)*

8. Rolled 1 and then 2, reported 4. (*Unjustified lie)*
9. Rolled 5 and then 2, reported 5. (*Honest)*
10. Rolled 4 and then 3, reported 5. (*Unjustified lie)*
11. Rolled 3 and then 4, reported 4. (*Justified lie)*
12. Rolled 2 and then 3, reported 3. (*Justified lie)*
13. Rolled 6 and then 2, reported 6. (*Honest)*
14. Rolled 3 and then 2, reported 3. (*Honest)*
15. Rolled 3 and then 2, reported 6. (*Unjustified lie)*
16. Rolled 4 and then 5, reported 5. (*Justified lie)*
17. Rolled 5 and then 3, reported 5. (*Honest)*
18. Rolled 1 and then 4, reported 4. (*Justified lie)*

6 Study 2 dependent variables.

1. To what extent do you believe that the reported (first) outcome is a lie?

2. If you would have been in this situation, to what extent do you believe you would have reported the same as this person?

3. I think the outcome of the third die roll is____

7 Study 1 descriptive statistics.

		Friend		Stranger	
	**Outcome**	*M*	*SD*	*M*	*SD*
Perceived lie	1	1.38	.091	1.58	1.06
	2	1.18	0.55	1.83	1.08
	3	1.26	0.64	2.33	1.42
	4	1.44	.072	2.9	1.65
	5	1.64	1.14	3.35	1.67
	6	1.69	1.15	3.75	1.91
Information seeking	1	2.37	1.89	2.94	1.90
	2	2.27	1.74	2.88	1.81
	3	2.18	1.58	2.84	1.64
	4	2.32	1.63	3.2	1.59
	5	2.39	1.67	3.53	1.87
	6	2.63	1.98	3.94	2.06

Figure 1 (Study 1)

Study 1: Perceived lie as a function of interaction partner and outcome.

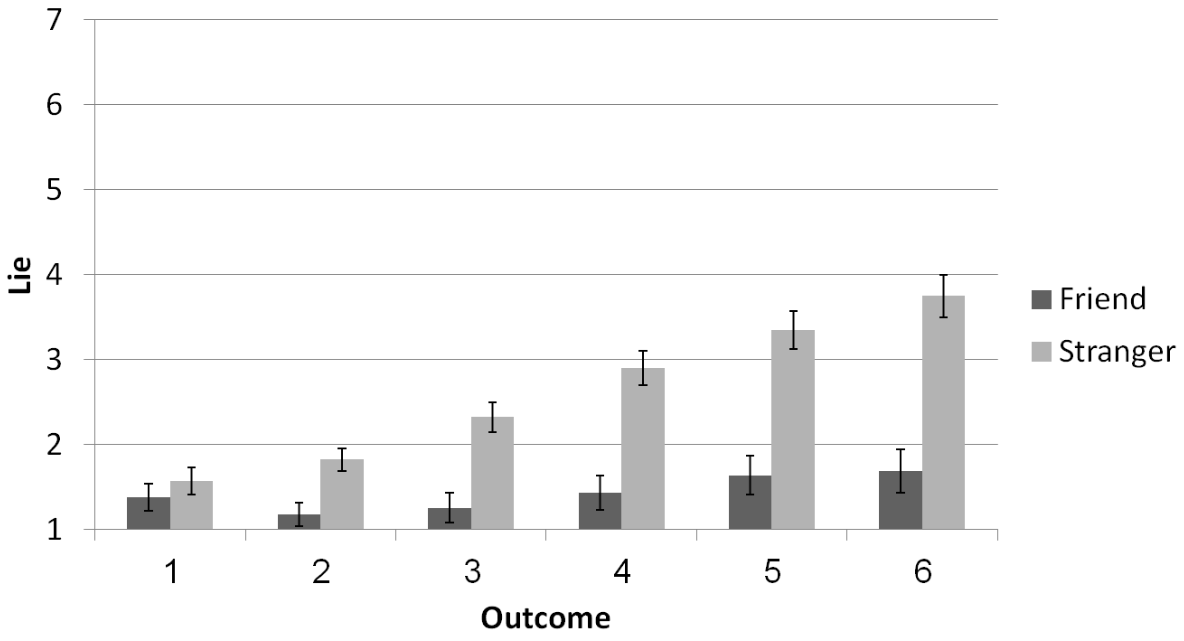

Figure S1. Perceived lie as a function of interaction partner and outcome.

Note. Error bars are +1 SE around the mean.

Figure 2 (Study 1)

Study 1: Information search about the second toss as a function of interaction partner and outcome.

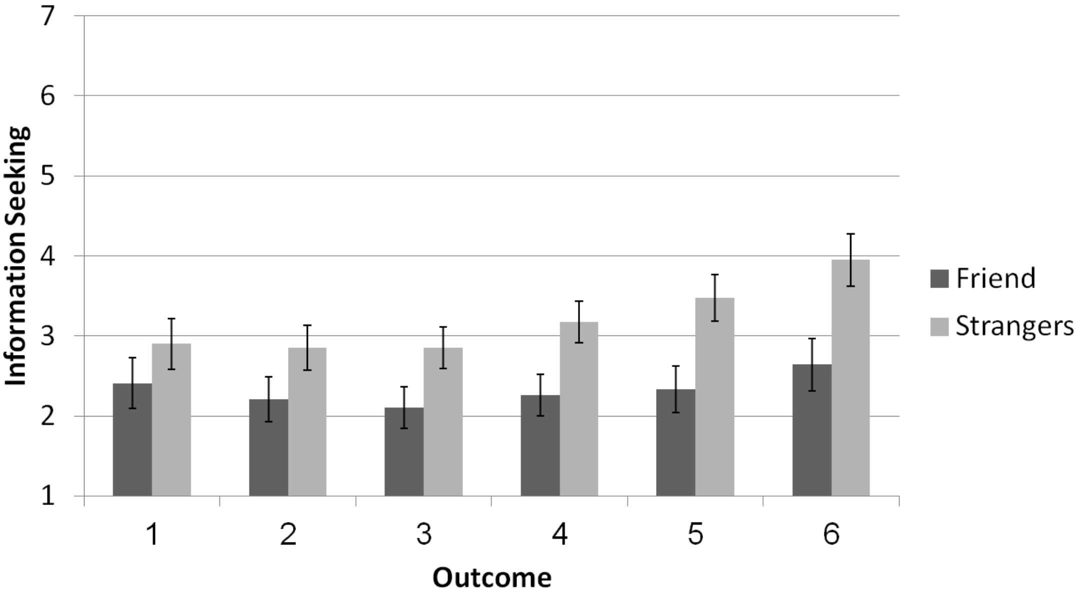

Figure S2. Information search about the second toss as a function of interaction partner and outcome.

Note. Information search was calculated by averaging participants' reports on a curiosity and wanting to know questions. Error bars are +1 SE around the mean.

Figure 3 (Study 2)

Study 2: Perceived lie as a function of the gap between real and reported outcome and available justification.

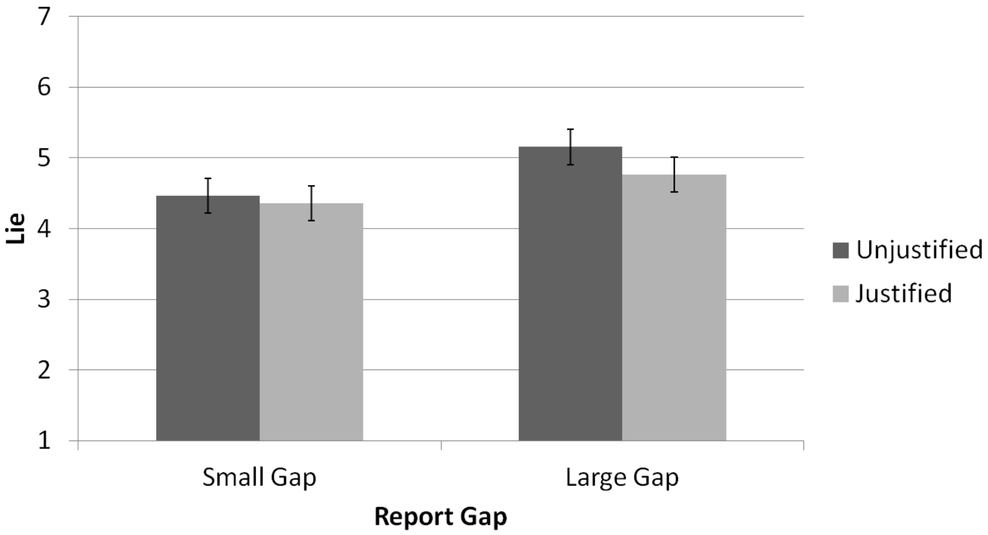

Figure S3. Perceived lie as a function of the gap between real and reported outcome and available justification.

Note. Results are across the both interaction partner conditions. Error bars are +1 SE

Figure 4 (Study 2)

Study 2: Participants' statements they would have done the same as the interaction partner, as a function of the gap between real and the interaction partner.

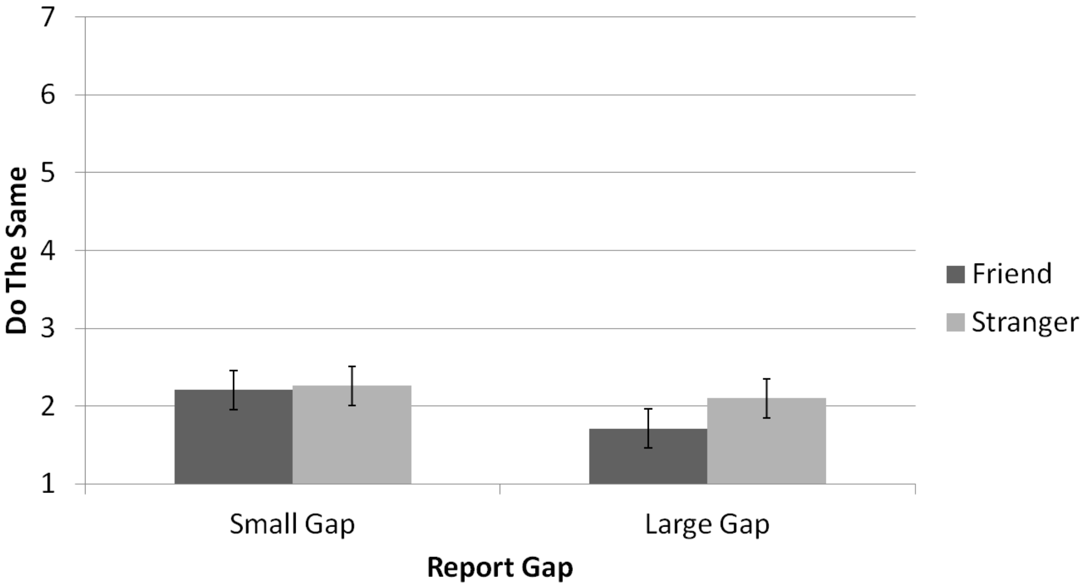

Figure S4. Participants' statements they would have done the same as the interaction partner, as a function of the gap between real and the interaction partner.

Note. Results are across the both justification conditions. Error bars are +1 SE around the mean.

Figure 5 (Study 2)

Study 2: Justifications generated as a function of the interaction partner and already available justification.

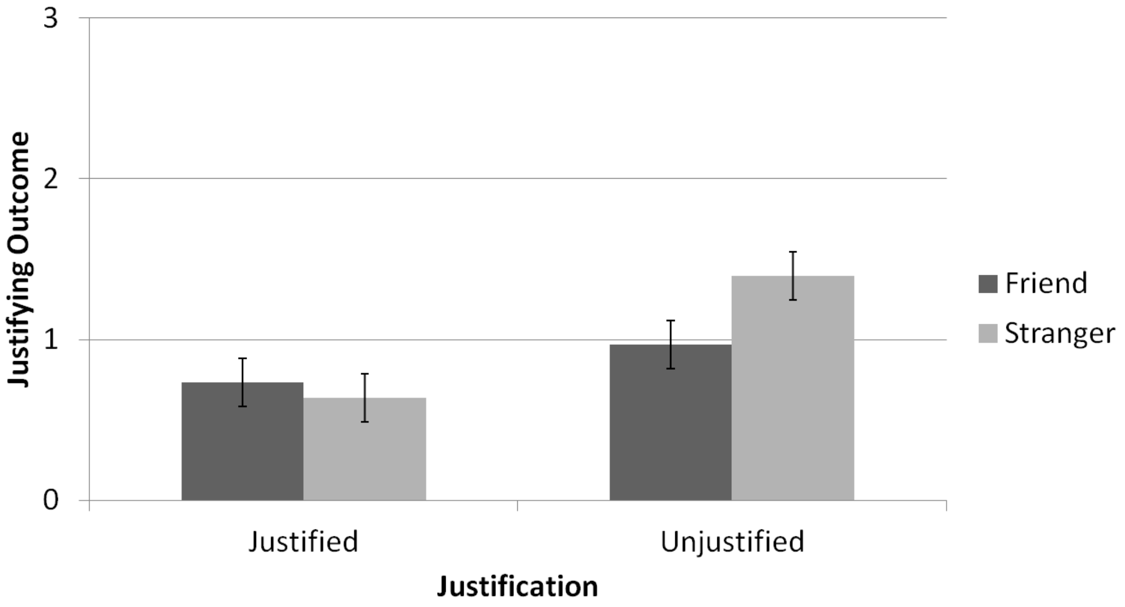

Figure S5. Justifications generated as a function of the interaction partner and already available justification.

Note. Results are across both report gap conditions. Justifications were measured by counting how many times each participants estimated the third die outcome to be equal to the reported first die outcome. Error bars are +1 SE around the mean
